# Isothiocyanates suppress the invasion and metastasis of tumors by targeting FAK/MMP-9 activity

**DOI:** 10.18632/oncotarget.19213

**Published:** 2017-07-12

**Authors:** Yun-Jeong Jeong, Hyun-Ji Cho, Fung-Lung Chung, Xiantao Wang, Hyang-Sook Hoe, Kwan-Kyu Park, Cheorl-Ho Kim, Hyeun-Wook Chang, Sang-Rae Lee, Young-Chae Chang

**Affiliations:** ^1^ Research Institute of Biomedical Engineering and Department of Medicine, Catholic University of Daegu School of Medicine, Daegu 705-718, Republic of Korea; ^2^ Department of Neural Development and Disease, Korea Brain Research Institute (KBRI), Daegu 701-300, Republic of Korea; ^3^ Department of Oncology, Lambardi Comprehensive Cancer Center, Georgetown University, Washington, DC 20057, USA; ^4^ National Institutes of Arthritis and Musculoskeletal and Skin Diseases, National Institutes of Health, Bethesda, MD 20892, USA; ^5^ Department of Biological Science, Sungkyunkwan University, Suwon, Kyunggi-Do 440-746, Republic of Korea; ^6^ College of pharmacy, Yeungnam University, Gyeongsan 701-947, Republic of Korea; ^7^ National Primate Research Center (NPRC), Korea Research Institute of Bioscience and Biotechnology (KRIBB), Chungbuk 28116, Republic of Korea

**Keywords:** isothiocyanates, metastasis, cancer invasion, MMP-9, FAK

## Abstract

Isothiocyanates, which are present as glucosinolate precursors in cruciferous vegetables, have strong activity against various cancers. Here, we compared the anti-metastatic effects of isothiocyanates (benzyl isothiocyanate (BITC), phenethyl isothiocyanate (PEITC), and sulforaphane (SFN)) by examining how they regulate MMP-9 expression. Isothiocyanates, particularly PEITC, suppressed 12-O-tetradecanoylphorbol-13-acetate (TPA)-induced MMP-9 activity and invasion in various cancer cell lines. By contrast, N-methyl phenethylamine, a PEITC analog without an isothiocyanate functional group, had no effect. A reporter gene assay demonstrated that BITC, PEITC, and SFN suppressed TAP-induced MMP-9 expression by inhibiting AP-1 and NF-κB in U20S osteosarcoma cells. All three compounds reduced phosphorylation of FAK, ERK1/2, and Akt. In addition, MMP-9 expression was downregulated by inhibiting FAK, ERK1/2, and Akt. Isothiocyanates-mediated inhibition of FAK phosphorylation suppressed phosphorylation of ERK1/2 and Akt in U2OS and A549 cells, along with the translocation of p65 and c-Fos, suggesting that isothiocyanates inhibit MMP-9 expression and cell invasion by blocking phosphorylation of FAK. Furthermore, isothiocyanates, abolished MMP-9 expression and tumor metastasis *in vivo* with the following efficacy: PEITC>BITC>SFN. Thus, isothiocyanates act as anti-metastatic compounds that suppress MMP-9 activity/expression by inhibiting NF-κB and AP-1 via suppression of the FAK/ERK and FAK/Akt signaling pathways.

## INTRODUCTION

Metastatic tumor may alter the surrounding stroma via direct cell contact or by secreting paracrine-soluble factors. Degradation of the extracellular matrix (ECM) and the basement membrane is an essential step in tumor metastasis [1]. Matrix metalloproteinases (MMPs) are ECM-degrading enzymes that mediate cell invasion and migration during tumor metastasis, wound healing, and angiogenesis [1, 2]. MMP-9 and MMP-2 are key enzymes that degrade type IV collagen, the major constituent of the basement membrane [3], and are mainly associated with tumor invasion, degradation of bone tissue, and angiogenesis [4, 5]. MMP-9 and MMP-2 have structural and catalytic similarities; however, whereas the activity of MMP-9 is increased by growth factors, cytokines, and 12-O-tetradecanoylphorbol-13-acetate (TPA), that of MMP-2 is not affected. MMP-9 is also modulated by transcription factors, including the transcription factor activator protein (AP)-1 (located at −79 and −533 bp) and nuclear factor (NF)-κB (−600 bp) [6]. Focal adhesion kinase (FAK) promotes secretion of MMP-9; conversely, inhibition of FAK reduces secretion of MMP-9 in carcinoma cells [7]. In addition, FAK promotes with tumor proliferation, apoptosis, adhesion and migration via accumulation and depolymerization of cytoskeleton proteins [8].

Isothiocyanates are naturally occurring substances in cruciferous vegetables (e.g., broccoli, cabbage, cauliflower, radishes, and watercress) formed from glucosinolate precursors [9]. They are a family of small organosulfur molecules characterized by the presence of an –N=C=S group and have antioxidant and anticancer properties. The compounds are effective against, breast, lung, colon, and prostate cancer [10–12]. Among the various isothiocyanates, benzyl isothiocyanate (BITC), phenethyl isothiocyanate (PEITC), and sulforaphane (SFN) show have a robust efficacy in preclinical cancer models [13]. They appear to act via diverse mechanisms, including induction of apoptosis and oxidative stress and inhibition of cell cycle progression [9, 14]. In addition, isothiocyanates regulate various signaling pathways and inhibit expression/activity of MMP-9 or MMP-2 [15, 16]. However, no study has examined and compared the anti-metastatic effects of different isothiocyanates *in vitro* and *in vivo*.

Therefore, the present study, examined the specific mechanisms by which BITC, PEITC, SFN, and N-methyl phenethylamine (NMPEA), a PEITC analog lacking an isothiocyanate functional group, downregulate MMP-9 expression in various cancer cells. We then compared the anti-tumor development and anti-metastatic effects of isothiocyanates *in vivo* by subcutaneous and tail vein injection into a mouse xenograft tumor model. We found that isothiocyanates harboring an –N=C=S group suppressed tumor metastasis by inhibiting FAK/MMP-9 activity and that the magnitude of the anti-tumor and anti-metastatic effects *in vivo* differed according to the isothiocyanate injected.

## RESULTS

### BITC, PEITC, and SFN abolish TPA-induced MMP-9 activity and expression in U2OS cells

**Figure 1:**
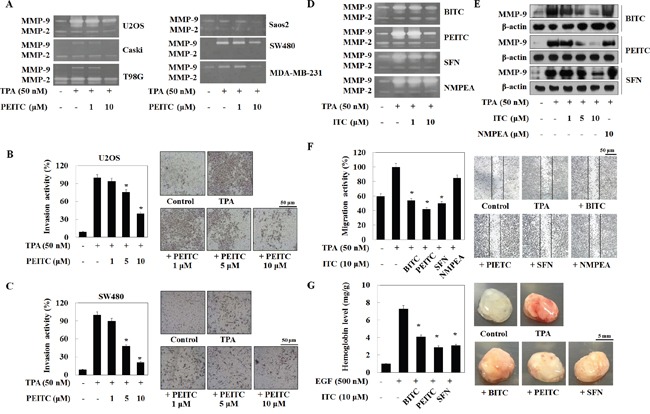
PEITC inhibits MMP-9 activity and tumor invasion of various cancer cells **(A)** Cells in serum-free medium were treated with PEITC followed by TPA and incubated for 24 h. MMP-9 activity was analyzed by a zymography assay. **(B** and **C)** Cells were loaded into the upper chamber of Matrigel-coated Transwells and treated with PEITC and cultured for 24 h. **(D** and **E)** U2OS cells were treated with TPA plus isothiocyanates for 24 h. MMP-9 activity and expression were analyzed in a zymography assay and by western blotting, respectively. β-actin was used as a control. **(F)** U2OS cells were cultured on a dish, scratched, and then treated with isothiocyanates followed by TPA for 24 h. Migrating cells were photographed by phase contrast microscopy. **(G)** C57BL/6N mice (n = 4) were injected subcutaneously with a mixture of Matrigel (500 μL) and U2OS cells (3 × 10^6^ cells), along with the indicated drugs. Mice were euthanized 7 days after implantation, and the Matrigel plugs were removed and photographed. The amount of hemoglobin in the Matrigel plugs was quantified using Drabkin's reagent. Data represent the mean ± S.E. of three independent experiments. *p<0.05 vs. TPA.

PEITC suppresses metastasis of human gastric cancer cells by inhibiting MMP-2 and MMP-9 [17]. Therefore, we first confirmed the inhibitory effects of PEITC on MMP-9 and MMP-2 in various cancer cells. Contrary to our expectations, PEITC suppressed both MMP-2 activity and TPA-induced MMP-9 in U20S, Caski, and T98G cells in a dose-dependent manner. PEITC inhibited the TPA-induced MMP-9 activity in Saos2, SW480, and MDA-MB-231 cells; however, MMP-2 activity was not detectable (Figure 1A). In addition, PEITC inhibited invasion of U2OS and SW480 cells was inhibited in a dose-dependent manner (Figure 1B and 1C), suggesting that the effects of PEITC on the cancer cell invasion are related to regulation of MMP-9 activity.

Because TPA most strongly induced secretion of MMP-9 by U2OS cells, we next compared the effect of different isothiocyanates on MMP-9 activity in U2OS cells. Zymography (Figure 1D) and western blot analysis (Figure 1E) revealed that TPA-induced MMP-9 activity and protein expression fell gradually upon exposure to BITC, PEITC, and SFN, but not upon exposure to NMPEA. A wound healing assay also showed that BITC, PEITC, and SFN, but nor NMPEA, suppressed migration of U2OS cells. BITC, PEITC, and SFN (at 10 μM) inhibited U2OS cell migration by 46%, 58%, and 50%, respectively (Figure 1F). In addition, isothiocyanates (1-30 μM) induced a ∼7% decrease in U2OS cell viability (Supplementary Figure 1), suggesting no significant cytotoxicity.

Because tumor angiogenesis correlates closely with MMP activity and metastasis [4, 5], we performed an *in vivo* Matrigel plug assay to assess angiogenesis in U2OS cells grafts implanted into C57BL/6N mice. In the presence of epidermal growth factor (EGF), U2OS cells induced new blood vessel formation, which was inhibited by BITC, PEITC, and SFN (Figure 1G). Taken together, these results suggest that the isothiocyanates abrogate MMP-9 activity and expression, thereby suppressing TPA-induced invasion and EGF-induced angiogenesis in U2OS model. Of note, PEITC suppressed MMP-9 activity to a greater extent than the other compounds.

### BITC, PEITC, and SFN inhibit the activity of AP-1 and NF-κB

**Figure 2:**
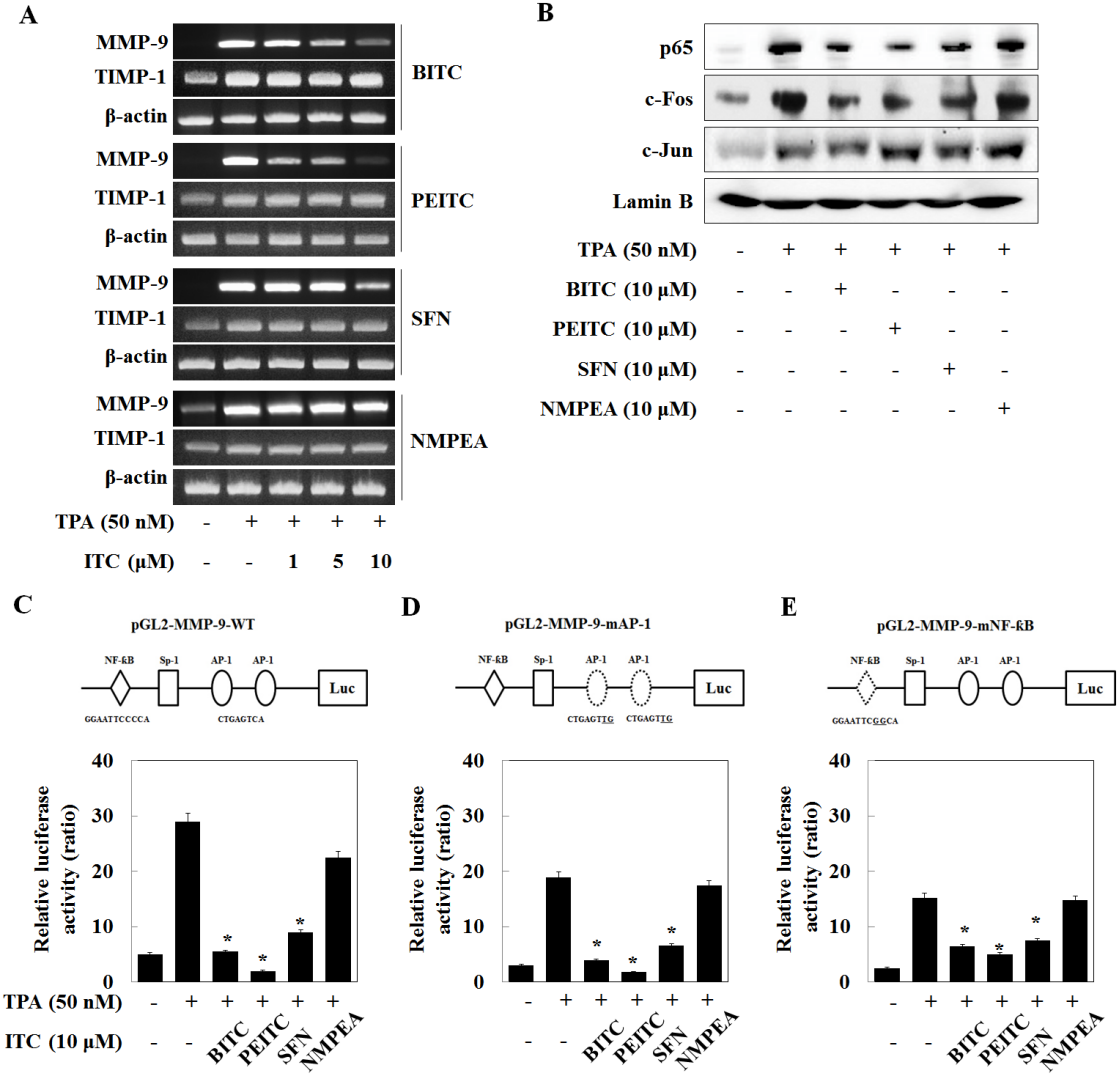
Isothiocyanates inhibit the activity of AP-1 and NF-κB in the MMP-9 promoter in U2OS cells **(A)** U2OS cells were treated with TPA and/or isothiocyanates for 24 h. Expression of mRNA encoding MMP-9 and TIMP-1 was then analyzed by RT-PCR. **(B)** U2OS cells were treated with TPA and isothiocyanates for 24 h, and the expression of p65, c-Fos, and c-Jun, and Lamin B (internal control) was analyzed by western blotting following nuclear extraction. **(C–E)** U2OS cells were transfected with the pGL2-MMP-9-WT, pGL2-MMP-9-mAP-1, and pGL2-MMP-9-mNF-κB reporter plasmids and then treated with TPA and isothiocyanates for 24 h. Relative luciferase activity in the cell extract was then measured.

RT-PCR was performed to further determine the mechanisms by which isothiocyanates downregulate MMP-9 protein expression. BITC, PEITC, and SFN all reduced the TPA-induced MMP-9 mRNA level in U2OS cells; however, isothiocyanates did not alter the level of mRNA encoding TIMP-1 (Figure 2A), an endogenous inhibitor of MMP-9 [18]. NMPEA did not alter the levels of mRNA encoding MMP-9 and TIMP-1, indicating that the sulfur-containing functional group of isothiocyanates directly inhibits MMP-9 transcription. Thus, we nest measured the expression of transcriptional elements in the MMP-9 gene, including AP-1 and NF-κB. Nuclear translocation of TPA-induced p65 (a NF-κB subunit) and c-fos (an AP-1 subunit) was suppressed by BITC, PEITC, and SFN, whereas that of c-jun (an AP-1 subunit) was not (Figure 2B).

In addition, to investigate how isothiocyanates affect the activity of the promoter regions of these transcription factors, cancer cells were transiently transfected with reporter constructs encoding the wild-type MMP-9 promoter (MMP-9-WT), or an MMP-9 promoter bearing mutations in AP-1-binding sites (MMP-9-mAP-1) or the NF-κB-binding site (MMP-9-mNF-κB). The promoter activity of MMP-9-WT, MMP-9-mAP-1, and MMP-9-mNF-κB was markedly suppressed by BITC, PEITC, and SFN, but not by NMPEA (Figure 2C–2E). In addition, the activity of MMP-9-mAP-1 was suppressed by BITC, PEITC, and SFN to a greater extent than that of MMP-9-mNF-κB. These results suggest that isothiocyanates suppress both AP-1- and NF-κB-driven MMP-9 expression, although NF-κB plays a more dominant role.

### BITC, PEITC, and SFN suppress the phosphorylation of ERK, Akt and FAK

**Figure 3:**
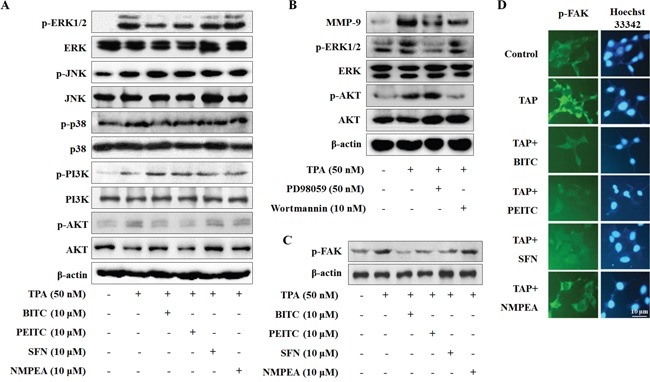
Isothiocyanates inhibit phosphorylation of ERK, Akt, and FAK in U2OS cells **(A** and **C)** U2OS cells were incubated with isothiocyanates and TPA for 24 h, and expression of the indicated proteins was determined by western blotting. **(B)** U2OS cells were treated with PD98059 or wortmannin for 24 h. Expression of MMP-9 and total and phosphorylated forms of ERK1/2 and Akt were measured by western blotting. β-actin is shown as a control. **(D)** Immunofluorescence microscopy analysis of changes in FAK phosphorylation after isothiocyanate treatment. U2OS cells were co-stained with an anti-p-FAK antibody and Hoechst 33342 and observed under a fluorescence microscope. Data are representative of three independent experiments.

Mitogen-activated protein kinase (MAPK) pathways and PI3K/Akt pathway are critical for TPA-induced MMP-9 in several cell types [19]. Thus, we examined the effect of isothiocyanates on MAPK and PI3K/Akt. Phosphorylation of extracellular signal-regulated kinase (ERK), JNK, p38 kinases, PI3K, and Akt increased upon exposure to TPA. BITC, PEITC, and SFN inhibited the phosphorylation of ERK1/2 and Akt, but not that of JNK, p38, or PI3K (Figure 3A). In addition, PD98059 (a MEK inhibitor) and wortmannin (a PI3K inhibitor) inhibited MMP-9 expression (Figure 3B), suggesting that BITC, PEITC, and SFN suppress expression of MMP-9 by inhibiting phosphorylation of ERK and Akt. Furthermore, we evaluated the effect of isothiocyanates on FAK phosphorylation. TPA increased FAK phosphorylation; however, TPA-induced FAK phosphorylation was suppressed by BITC, PEITC, and SFN (Figure 3C). This result was confirmed by immunofluorescence analysis. Cell nuclei were stained with Hoechst 33342, and phosphorylated FAK was labeled with FITC. Phosphorylation of FAK was inhibited by BITC, PEITC, and SFN, but not by NMPEA (Figure 3D). These results suggest that isothiocyanate-inhibited MMP-9 is caused by inhibition of FAK phosphorylation.

### BITC, PEITC, and SFN inhibit MMP-9 expression by suppressing the phosphorylation of FAK

**Figure 4:**
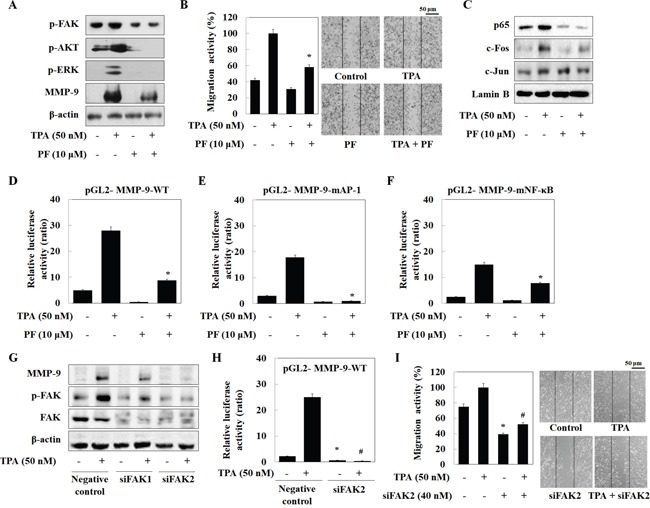
Isothiocyanates inhibit MMP-9 activity/expression and tumor invasion by suppressing phosphorylation of FAK in U2OS cells **(A** and **C)** U2OS cells were incubated with isothiocyanates and TPA for 24 h, and expression of the indicated proteins was determined by western blotting. **(B)** U2OS cells were plated onto a dish, scratched with a yellow pipette tip, and treated with PF573228 and TPA for 24 h. Migrating cells were photographed by phase contrast microscopy. **(D–F)** U2OS cells were transfected with the pGL2-MMP-9-WT, pGL2-MMP-9-mAP-1, and pGL2-MMP-9-mNF-κB reporter plasmids. The cells were then cultured with TPA and PF573228. Relative luciferase activity in the cell extract was then measured. **(G)** U2OS cells were transfected with negative control siRNA, siFAK1, or siFAK2, and then treated with TPA. Protein expression was determined by western blotting. **(H)** U2OS cells were transfected with the pGL2-MMP-9-WT reporter plasmid and siFAK2, and then treated with TPA for 24 h. Relative luciferase activity in the cell extract was then determined. **(I)** Cells were scratched, transfected with siFAK2, and then treated with TPA for 24 h. Migrating cells were photographed by phase contrast microscopy. Data represent the mean ± S.E. of three independent experiments. *p < 0.05 *vs*. control and #*p* < 0.05 *vs*. TPA.

To confirm involvement of FAK in isothiocyanate- inhibited MMP-9 transcription, we treated cells with PF573228 (a FAK inhibitor). PF573228 suppressed TPA-induced phosphorylation of FAK, Akt, and ERK in U2OS cells, along with MMP-9 expression (Figure 4A) and cell migration (Figure 4B). In addition, we asked whether FAK inhibits AP-1 and NF-κB expression mediated by isothiocyanates. The results were similar to those presented in Figure 2B; PF573228 suppressed TPA-induced nuclear translocation of p65 and c-Fos, but not that of c-Jun (Figure 4C). In the results of a reporter gene assay, PF573228 also inhibited the activity of MMP-9-WT, MMP-9-mAP-1, and MMP-9-mNF-κB. As expected, the activity of MMP-9-mAP-1 was suppressed to a greater extent than that of MMP-9-mNF-κB (Figure 4D–4F), indicating that FAK plays a key factor in the isothiocyanates-inhibited MMP-9 expression.

Furthermore, we transiently transfected U2OS cells with two siRNAs targeting FAK. Both caused a marked reduction in phosphorylation of FAK and MMP-9 (Figure 4G). Because siFAK2 inhibited MMP-9 activity to a greater extent than siFAK1, we next transiently transfected U2OS cells with siFAK2 and a reporter gene. MMP-9-WT activity was abolished by siFAK2 (Figure 4H). Transfection of siFAK2 inhibited U2OS cell migration both in the presence and absence of TPA (Figure 4I). These results suggest that isothiocyanates inhibit TPA-induced MMP-9 expression by blocking MMP-9 promoter activity via suppression of FAK phosphorylation.

### BITC, PEITC, and SFN prevent TPA-induced MMP-9 activity and cell migration in various cancer cells

**Figure 5:**
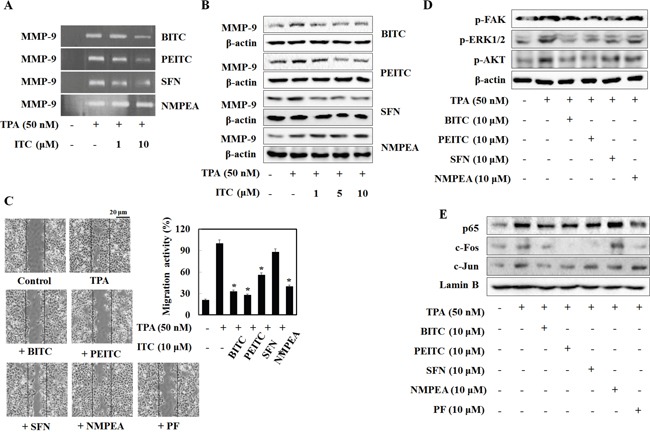
Isothiocyanates inhibit MMP-9 activity and tumor invasion by suppressing phosphorylation of FAK in A549 cells **(A, B**, and **D)** A549 cells were treated with TPA and isothiocyanates for 24 h. MMP-9 activity and expression were then analyzed in a zymography assay and by western blotting, respectively. β-actin is shown as a control. **(C)** A549 cells were plated onto a dish, scratched, and then treated with isothiocyanates followed by TPA for 24 h. Migrating cells were photographed by phase contrast microscopy. **(E)** A549 cells were treated with TPA, isothiocyanates, or PF573228 for 24 h, and expression of p65, c-Fos, c-Jun, and Lamin B (internal control) was analyzed by western blotting following nuclear extraction. Data represent the mean ± S.E. of three independent experiments. *p < 0.05 *vs*. TPA.

Experiments using heterotopic grafts under the mouse skin show that U2OS cells are generally non-tumorigenic and do not readily form lung metastases [20, 21]. Among the commonly used xenograft metastasis models, tail vein injection of A549 cells results in the highest rates of metastasis (90–100%) [22]. Therefore, before using A549 cells in a tumor xenograft model, we first examined the effects of isothiocyanates on TPA-induced MMP-9 activity in A549 lung cancer cells. As expected, BITC, PEITC, and SFN suppressed TPA-induced MMP-9 activity and expression (Figure 5A and 5B). PEITC inhibited MMP-9 activity and expression to a greater extent than the other compounds, whereas NMPEA had no effect. A wound healing assay showed that BITC, PEITC, and SFN (at 10 μM) inhibited migration of A549 cells (Figure 5C) by 33%, 28%, and 56%, respectively. The inhibitory effects of isothiocyanates on MMP-9 and cell migration were also apparent in MDA-MB-231 and SW480 cells (Supplementary Figure 2), suggesting that the ability of isothiocyanates to inhibit migration is dependent on the sulfur-containing functional group, rather on the cancer cell line itself.

To confirm the mechanism by which isothiocyanates inhibit MMP-9 expression in A549 cells, we asked which kinases and transcription factors of MMP-9 (FAK, ERK, Akt, p65, c-fos, and c-Jun) were inhibited upon isothiocyanate treatment of U2OS cells. Similar to the results for U2OS cells, we found that BITC, PEITC, and SFN reduced TPA-induced phosphorylation of FAK, ERK1/2, and Akt in A549 cells (Figure 5D). BITC and PEITC also caused a significant reduction in TPA-induced nuclear translocation of p65, c-Fos, and c-Jun; however, SFN and PF573228 suppressed the nuclear translocation of p65 and c-Fos, but not c-Jun. Although the inhibitory effect of BITC and PEITC on c-fos nuclear translocation was different from that observed in U2OS cells, the results indicate that isothiocyanates suppress MMP-9 in both A549 and U2OS cells by inhibiting transcriptional activity.

### BITC, PEITC, and SFN inhibit xenograft tumor growth in nude mice

**Figure 6:**
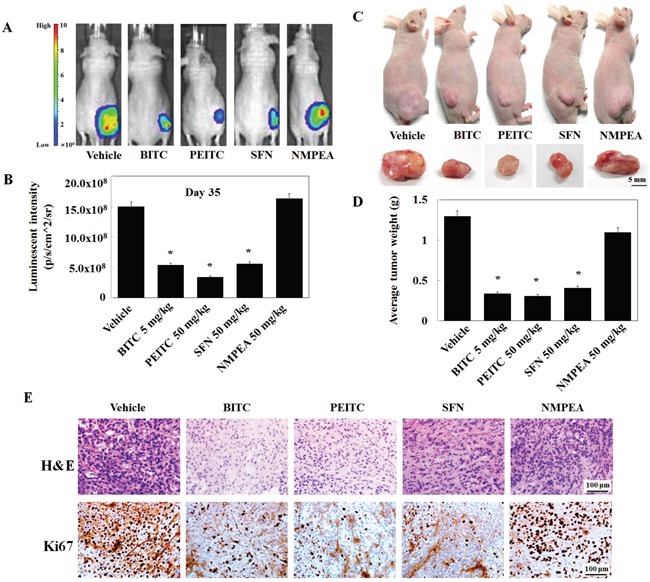
Isothiocyanates inhibit tumor growth in a phoenix A-transfected A549 cell xenograft mouse model **(A** and **B)** Mice were inoculated subcutaneously into the right flank with 1 × 10^7^ phoenix A-transfected A549 cells. After the formation of palpable tumors (∼5 mm by Day 14), the mice were randomized into five groups (n = 4/group) and injected intraperitoneally (or not) with 5 mg/kg/100 μL BITC, PEITC, or SFN. Injections were administered daily. Mice were sacrificed on Day 35, and tumor size was measured. Bioluminescence imaging of tumor xenografts was performed following intraperitoneal injection of D-luciferin into mice previously injected with phoenix A-transfected A549 cells. Tumors were imaged using an *in vivo* imaging system. **(C)** Inset images show the sizes of representative tumors. **(D)** Average tumor weight in vehicle- and isothiocyanate-treated mice. Data represent the mean ± S.E. *p < 0.05 *vs*. vehicle. **(E)** Tumors were fixed, sectioned, and stained with hematoxylin and eosin (H&E) to examine tumor cell morphology. An anti-Ki67 antibody was used to assess tumor cell proliferation.

Finally, to investigate whether isothiocyanates inhibit tumor growth *in vivo*, we generated a mouse xenograft tumor model using phoenix A-transfected A549 cells. Prior to the *in vivo* experiment, we assessed the effect of isothiocyanates on phoenix A-transfected A549 cells *in vitro*. As in U2OS cells, BITC, PEITC, and SFN inhibited TPA-induced MMP-9 activity and expression in phoenix A-transfected A549 cells (Supplementary Figure 3). The right flank of mice was then injected subcutaneously with phoenix A-transfected A549 cells (1 × 10^7^). Once the tumors became palpable (mean diameter, 5 mm), the mice were injected intraperitoneally with each isothiocyanate compound for 5 weeks.35 post-injection, the animals were sacrificed and the final tumor weight and volume were measured. Mice engrafted with vehicle and those treated with 50 mg/kg NMPEA displayed approximately 15.0 × 10^8^ and 16.3 × 10^8^ Luminescent intensity (Figure 6A and 6B). However, mice injected with 5 mg/kg BITC, 50 mg/kg PEITC, and 50 mg/kg SFN displayed approximately 5.4 × 10^8^, 3.4 × 10^8^ and 5.6 × 10^8^ Luminescent intensity. Isothiocyanates also led to a reduction in tumor weight when compared with vehicle or NMPEA; the maximum tumor size in PEITC-treated mice was considerably smaller than that in the other groups (Figure 6C and 6D). Ki67 expression was also measured by immunohistochemical analysis of tumors treated with isothiocyanates. Ki67, a marker of cell proliferation, was suppressed by isothiocyanates but not by the vehicle (Figure 6E). These results indicate that isothiocyanates block proliferation of A549 cells and subsequent tumor development, and that the most effective isothiocyanate that inhibited tumor development was BITC.

**Figure 7:**
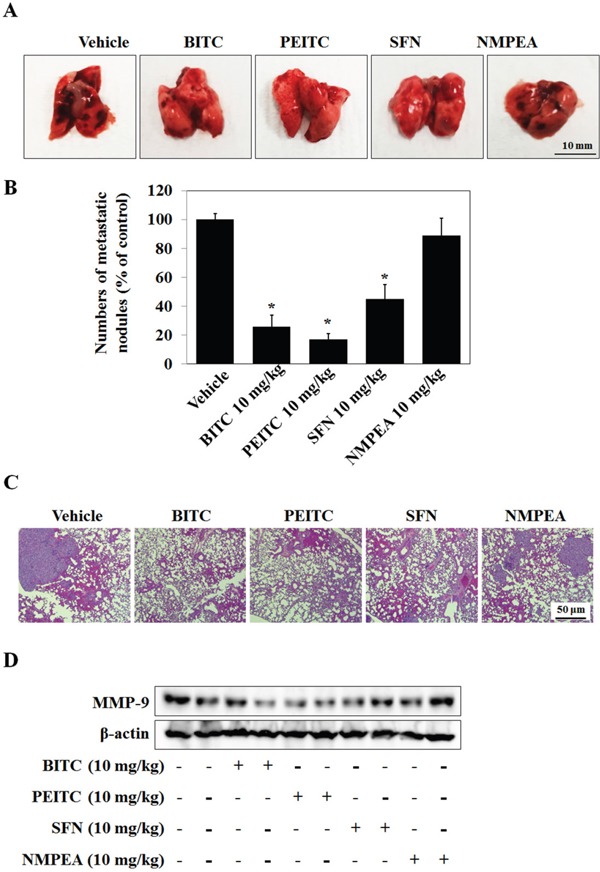
Isothiocyanates inhibit tumor metastasis in an A549 xenograft mouse model **(A)** BALB/c nude mice (n = 5 per group) received a tail vein injection of A549 cells. The number of surface lung nodules was then quantified statistically. **(B)** The number of metastatic lung nodules in individual mice was counted under a dissection microscope. Data represent the mean ± S.E. *p < 0.05 *vs*. vehicle. **(C)** Representative images of surface lung nodules in sections stained with H&E. **(D)** Expression of MMP-9 in vehicle- and isothiocyanate-treated tumor tissue was determined by western blotting. β-actin is shown as a control. Data are representative of three independent experiments.

Furthermore, we examined the anti-metastatic effects of isothiocyanates through injecting A549 cells into the tail veins of nude mice. Macroscopic and histological observations revealed lung metastasis in the vehicle group. Significantly reduced number of metastatic lung nodules were observed in the 10 mg/kg BITC- and 10 mg/kg PEITC-injected groups and 10 mg/kg SFN also inhibits lung cancer metastasis compared with vehicle and NMPEA group (Figure 7A and 7B). Representative images of tissue sections stained with hematoxylin & eosin (H&E) in Figure 7C. The vehicle and NMPEA groups showed high density of tumor cells, whereas expression was much lower in the BITC, PEITC, and SFN groups. In addition, BITC, PEITC, and SFN suppressed MMP-9 expression in lung tissue (Figure 7D). In results of H&E staining and western blot analysis, PEITC was the most effective at reducing both the number of metastatic lung nodules and MMP-9 expression, suggesting that the anti-metastatic effects of isothiocyanates are associated with inhibition of MMP-9 expression.

## DISCUSSION

Dietary intake of cruciferous vegetables may reduce the risk of various types of cancer; this may be due to the presence of phytochemicals such as isothiocyanates in these foods [23]. Here, we demonstrated the anti-metastatic effects of isothiocyanates (BITC, PEITC, and SFN) both *in vitro* and *in vivo*. We first examined the effect of isothiocyanates on MMP-9 activity, which plays a critical role in tumor metastasis [24], and cancer cell migration. BITC, PEITC, and SFN suppressed both MMP-9 activity and migration of cancer cells regardless of MMP-2 activity status. These results suggest that inhibiting MMP-9 activity is more important than inhibiting MMP-2 activity in terms of the anti-metastatic effects of isothiocyanates. In general, compounds containing electrophilic carbon structures (−N=C=S) appear to have stronger anti-proliferative activity [25]. We used a PEITC analog, NMPEA, which lacks the electrophilic carbon structure, to demonstrate the effects of this electrophilic moiety on cancer cell metastasis. The relative potency with which isothiocyanates inhibited both MMP-9 activity and cancer cell migration showed the following ranking: PEITC>BITC>SFN.

Isothiocyanates affect various cellular pathways, including apoptosis, MAPK, oxidative stress, and cell cycle progression [9, 14]. We found that all of three isothiocyanates inhibited translocation of p65 and c-fos, as well as the promoter activity of MMP-9-WT, MMP-9-mAP-1, and MMP-9-mNF-κB. AP-1 and NF-κB induced expression of MMP-9 via the MAPK or PI3K/Akt pathway [26, 27]. Isothiocyanates inhibited phosphorylation of ERK and Akt in U20S cells, but did not affect phosphorylation of JNK, p38, and PI3K. PD98059 and Wortmannin also reduced MMP-9 expression, suggesting that BITC, PEITC, and SFN suppress MMP-9 in U20S cells by inhibiting phosphorylation of ERK and Akt. However, BITC suppresses expression of PI3K/Akt in pancreatic tumors [28], and SFN reduces phosphorylation of PI3K/Akt in ovarian cancer cells [29]. In C6 glioma cells, BITC, PEITC, and SFN inhibited the TPA-induced JNK phosphorylation without affecting phosphorylation of ERK or p38 [7]. Therefore, the inhibitory effects of isothiocyanates on MAPK or PI3K/Akt phosphorylation depend on the cell lines.

FAK contributes to a cellular phenotype that remodels the ECM via expression and release of MMP-2 and MMP-9 [30]. Isothiocyanates and siFAK blocked the promoter activity of MMP-9 and cell migration. Previous studies showed that activation of AP-1 and NF-κB is inhibited in FAK mutated cells [31, 32]. Our results observed that promoter activity of MMP-9-mAP-1 and MMP-9-mNF-κB were suppressed by isothiocyanates and the FAK inhibitor. FAK also regulates phosphorylation of ERK and Akt, and the FAK-ERK and FAK-Akt signaling pathway induces MMP-9 expression in several cancer cell lines [33, 34]. We found that isothiocyanates reduced the phosphorylation of FAK, and that pharmacological inhibition of FAK suppressed cell migration, MMP-9 expression, and Akt and ERK phosphorylation. These results suggest that isothiocyanates inhibited MMP-9 through FAK/ERK/Akt- and NF-κB/AP-1-dependent mechanisms.

Our *in vivo* studies reveal that different isothiocyanates have different effects on tumor development and metastasis. BITC was the most effective at inhibiting tumor development, whereas PEITC was the most effective at inhibiting metastasis. SFN was the least potent; however, NMPEA had no effect at all, suggesting that the –N=C=S group plays an essential role in mediating the anticancer effects of isothiocyanates. In the xenograft models, BITC, PEITC, and SFN inhibited MMP-9 expression in tumors (Supplementary Figure 3; PEITC>BITC>SFN). This suggests that the inhibitory effects of isothiocyanate on MMP-9 expression are closely associated with their anti-metastasis effects. In addition, previous studies show that proliferation of A549 cells is reduced significantly by isothiocyanates in the order BITC > PEITC > SFN [35], suggesting that tumor development is related to cell proliferation.

**Figure 8:**
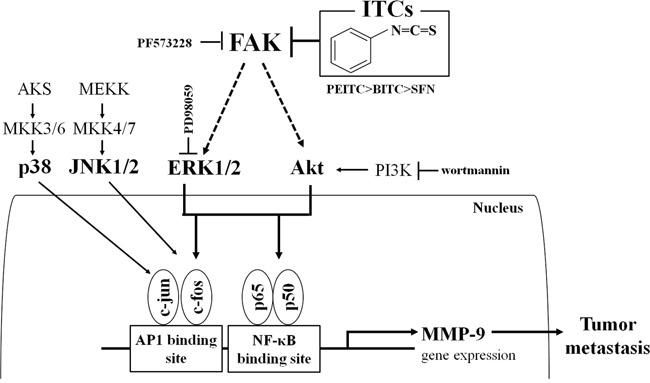
Schematic model for suppression of MMP-9 activity/expression by isothiocyanates Isothiocyanates exert anti-metastatic activity by inhibiting MMP-9 with the following efficacy: PEITC>BITC>SFN. They suppress MMP-9 activity/expression by inhibiting NF-κB and AP-1 via suppression of the FAK/ERK and FAK/Akt signaling pathways.

In summary, isothiocyanates inhibit tumor growth and metastasis by suppressing MMP-9 activity/expression, an effect achieved by blocking the FAK/ERK/Akt and NF-κB/AP-1 pathways (Figure 8). Isothiocyanates abolished MMP-9 expression and tumor metastasis *in vivo*. To the best of our knowledge, this is the first study to demonstrate that isothiocyanates exert anti-metastatic activity by inhibiting MMP-9 and to rank the anticancer activity of these compounds (PEITC>BITC>SFN) both *in vitro* and *in vivo*.

## MATERIALS AND METHODS

### Cells culture and materials

MDA-MB-231 (breast adenocarcinoma), Caski (cervical carcinoma), A549 (lung adenocarcinoma epithelial cell), and U2OS (osteosarcoma) cell lines were obtained from the American Type Culture Collection (Rockville, MD, USA). SW480 (colon adenocarcinoma), T98G (brain glioblastoma multiform) and DU145 (prostate carcinoma) were obtained from prof. Chung's Lab. of Georgetown University. The culture medium used in the experiments was Dulbecco's modified Eagle's medium (DMEM; Gibco, Grand Island, NY, USA), containing 10% FBS, 20 mM HEPES, and 100 μg/ml gentamicin. Isothiocyanates (ITC; BITC (benzyl ITC), PEITC (phenethyl ITC), SFN (sulforaphane), and NMPEA (N-methyl phenethylamine)).

### Cell invasion assay

Cell invasion assay was carried out as previously reported [19] with slight modifications. The upper chamber of a trans-well insert (Corning Costar, Cambridge, MA) was coated with 30 μl of a 1:2 mixture of matrigel:PBS. The cells were plated on the matrigel-coated the upper chamber. The lower chamber was filled DMEM with various concentrations of PEITC. Cells in the chamber were incubated for 24 h at 37°C and cells that had invaded the lower surface of the membrane were fixed with methanol and stained with hematoxylin and eosin. Random fields were counted by light microscopy under a high power field.

### Wound healing assay

Wound healing assay was carried out as previously reported [36]. Cells were scratched with a yellow tip to create a wound, and cells were then washed twice with serum-free culture media to remove floating cells. Media were then replaced with fresh medium. Cells were subjected to the indicated treatment for 24 h, and cells migrating from the leading edge were photographed.

### Gelatin substrate gel zymography assay

Zymography was performed as previously reported [19]. The cells were plated at dishes, and incubated. Fresh serum-free medium was added to each dish, followed by further culturing for 24 h. The resultant supernatant was subjected to SDS-PAGE in 10% polyacrylamide gels that were copolymerized with 1 mg/ml of gelatin. After the electrophoresis runs, the gels were washed with 2.5% Triton X-100, and incubated for 24 h at 37°C in the buffer containing 5 mM CaCl_2_ and 1 μM ZnCl_2_. The gels were stained with Coomassie Brilliant Blue (0.25%) (Bio-Rad, California, USA). Proteolytic activity was evidenced as clear bands against the blue background of the stained gelatin.

### Western blot analysis

Total cell lysates were prepared by lysis buffer [50 mM Tris, 150 mM NaCl, 5 mM EDTA, 1 mM DTT, 0.5% NP-40, 100 μM phenylmethylsulfonyl fluoride, 20 μM aprotinin, and 20 μM leupeptin, adjusted to (pH 8.0)]. Total proteins were electrotransferred to Immobilon-P membranes (Millipore Corporation, Bedford, MA). Detection of specific proteins was carried out with an enhanced chemiluminescence Western blotting kit, following the manufacturer's instructions (Amersham, NJ, USA). To determine the activations of NF-κB and AP-1, nuclear extracts of cells were subjected to describe as previous reported [37].

### Reverse transcription-polymerase chain reaction (RT-PCR)

Total RNA was extracted using TRIzol (Invitrogen, USA), according to the manufacturer's instructions. Reverse transcription was carried out using a commercial kit (Superscript II RNase H-reverse transcriptase, Invitrogen) and total RNA 1 μg from U2OS cells, according to the manufacturer's protocol. Amplified products were resolved by 1.3% agarose gel electrophoresis and visualized by staining with ethidium bromide. We quantified the actual MMP-9 mRNA level by using Image Lab™ software (version 4.1; Bio-rad) The sequences of the primers were as follow: for MMP-9, 5′-cggagcacggagacgggtat-3′ (forward) and 5′-tgaaggggaagacgcacagc-3′ (reverse); for TIMP-1, 5′-ctgttgttgctgtggctgata-3′ (forward) and 5′-ccgtccacaagcaatgagt-3′ (reverse); for β-actin, 5′-caagagatggccacggctgct-3′ (forward) and 5′-tccttctgcatcctgtcggca-3′ (reverse). FAK1 siRNA (5′-ccaacaaacatttagacaa-3′) and FAK2 siRNA (5′-ccaggtttactgaacttaa-3′) were purchased from Genolution Pharmaceuticals, Inc. (Seoul, Kore).

### Matrigel plug assay

C57BL/6N mice were purchased from Samtako (Osan, Korea) and maintained in pathogen-free conditions. Mice housed four per cage in a room maintained at a constant temperature (25°C) in a light:dark 12:12 h schedule. Mice were fed a normal diet for 24 h. Aliquots of U2OS cell (3×10^6^ cells) were mixed with 0.5 ml of matrigel in the presence or absence of epidermal growth factor (EGF) (500 ng/ml) and isothiocyanates. Immediately, the mixture was subcutaneously injected into mice. The mice were sacrificed when tumors were visible, and the matrigel plugs were carefully separated from adjacent tissue and removed. Hemoglobin content was determined using Drabkin's reagent kit (Sigma Chemicals), as described previously [37]. All surgical and experimental procedures used in this study were approved by the Institutional Review Board Committee at Daegu Catholic University Medical Center which conforms to the US National Institutes of Health guidelines for care and use of laboratory animals.

### Plasmids transfection and luciferase reporter gene assay

The MMP-9 promoter contains cis-acting regulatory elements for transcription factors that include two AP-1 site (located at −79 bp and −533 bp) and an NF-κB site (located at −600 bp). MMP-9 wild type (pGL2-MMP-9WT), AP-1 site-mutated MMP-9 (pGL2-MMP-9mAP-1-1), and NF-κB site-mutated MMP-9 (pGL2-MMP-9mNF-κB) luciferase promoter constructs were used in transient transfection assays as described previously [38]. Cells were plated onto 35 mm dishes at a density of 2×10^5^ cells and allowed to grow overnight. The cells were then cotransfected with 2 μg of various plasmid constructs and 1 μg of the pCMV-β-galactosidase plasmid for 24 h by the Promega transfection activities were determined using commercial kits (Promega, Madison, WI, USA), according to manufacturer's protocols. Luciferase activity was calculated as luciferase activity normalized with β-galactosidase activity in each cell lysate.

### siRNA transfection

Cells were separated and total of 2×10^5^ cells were plated into 6-well plates at 37°C in a humidified atmosphere of 5% CO_2_ for 24 h. The cells grew to 50–60% confluency within the 24 h. For transfection experiments with siRNA directed against FAK (Santa Cruz Biotechnology, CA, USA), the medium for U2OS cells was changed to fresh DMEM, and cells were transfected for 24 h with 10 μM of siRNA by Trans IT-TKO (Mirusbio, Madison, WI) according to the manufacturer's instruction. After 24 h, the medium was changed to fresh serum-free DMEM, cells were treated with TPA or 24 h and total protein in the cells was extracted for Western blot.

### Immunofluorescence microscopy

Cells were cultured and treated on poly-_L_-lysine-coated coverslips before being fixed in 100% ethanol for 1 min at room temperature. After two washes with PBS, cells were permeabilized with 0.2% w/v Triton X-100 in PBS for 5 min, and then blocked with 10% v/v normal goat serum in PBS for 1 h in a humidified chamber. Cells were then incubated with primary antibodies (1:100, diluted in PBS containing 2% v/v normal goat serum) for 1 h. The cells were then washed three times before being incubated with FITC, tetramethylrhodamine isothiocyanate-conjugated secondary antibody 1 h, and three times were washed with PBS. Then, cells were incubated with Hoechst 33342 for 3 min at room temperature. Finally, the cells were washed five times with PBS containing 0.05% v/v Tween-20 and 1% w/v BSA. Coverslips were mounted and scanned on a fluorescence microscope.

### Tumor xenograft model

Animal model were in accordance with the guide-lines issued by the Institutional Animal Care and use committee of the Kyungpook National University School of Medicine. BALB/c nude mice were purchase from Lab Animal (Seoul, Korea) and maintained in pathogen-free conditions. The mice were inoculated with 1×10^7^ cells of phoenix A-transfected A549 cells subcutaneously into the right flank of mice. After the formation of palpable tumors (∼5 mm by day 14), the mice were randomized into five condition (n=4/group). The mice were treated with or without intraperitoneal injection of 5 mg/kg/100 μl per mouse BITC and, 50 mg/kg/100 μl per mouse PEITC, and SFN every day. Phoenix A-transfected A549 cells were visualized in the animal after intraperitoneal injection of 50 mg/kg luciferin (PerkinElmer, MA, USA) by bioluminescence imaging using *in vivo* image system (IVIS) (LAC, DGMIF). Mice were sacrificed and the on day 35, and tumor sizes were determined. Tissues were processed for sectioning, H&E staining, immunohistochemistry. We wish to thank that this animal research was supported by a technology & equipment of LAC (Laboratory Animal Center) in DGMIF (Daegu-Gyeongbuk Medical Innovation Foundation).

### Metastasis of tumor xenograft model

BALB/c nude mice (n = 5/group) received a tail vein injection of 50 μL RPMI medium (vehicle) or 1 × 10^6^/100 μL lung tumor cells on Day 0. The mice were then treated 10 mg/kg/100 μl per mouse BITC, PEITC, SFN, or NMPEA every day. 8 weeks later, the injection, lung tissues were processed for sectioning, H&E staining, western blot analysis. Metastatic lung nodules were counted and confirmed by examination of H&E-stained sections under a dissecting microscope.

### Statistical analysis

All *in vitro* results are representative of at least three independent experiments performed in triplicate. *P<0.05, statistically significant between experimental and control values. Significance of differences between experimental and control values was calculated using ANOVA with Newman-Keuls multicomparison test.

## SUPPLEMENTARY MATERIALS FIGURES


