# Supplementary material for: Isothiocyanates suppress the invasion and metastasis of tumors by targeting FAK/MMP-9 activity
Source: Oncotarget. 2017 Jul 12;8(38):63949–62. doi: 10.18632/oncotarget.19213 (PMC5609975; doi:10.18632/oncotarget.19213)
Supplement: Supplementary file 1 [file oncotarget-08-63949-s001.pdf]

Isothiocyanates suppress the invasion and metastasis of tumors by targeting FAK/MMP-9 activity

SUPPLEMENTARY MATERIALS

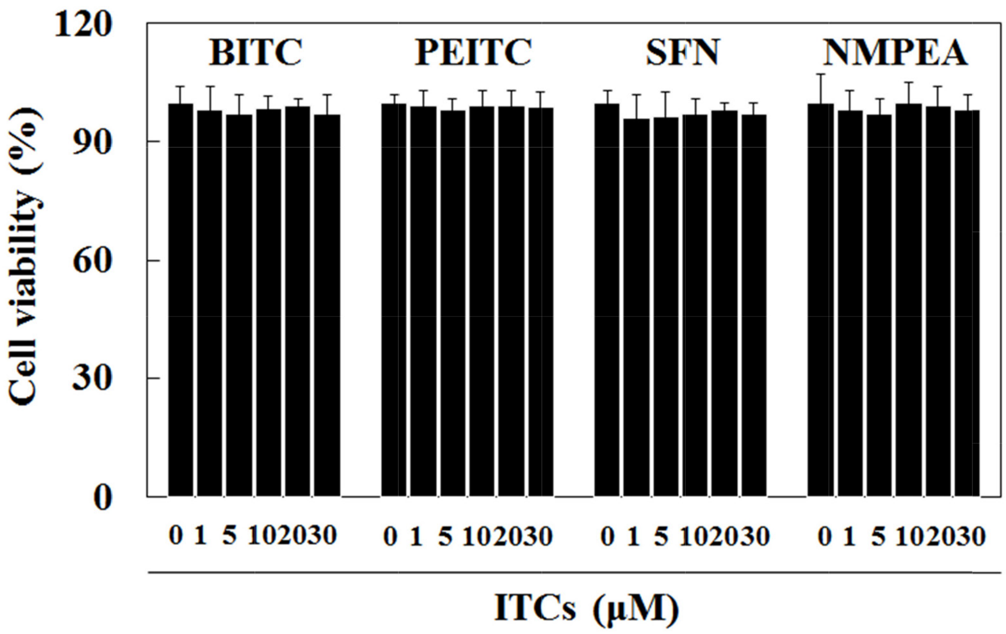

Supplementary Figure 1: U2OS cells were treated with the indicated concentrations of isothiocyanates for 24h. Cell viability was determined by an MTT assay.

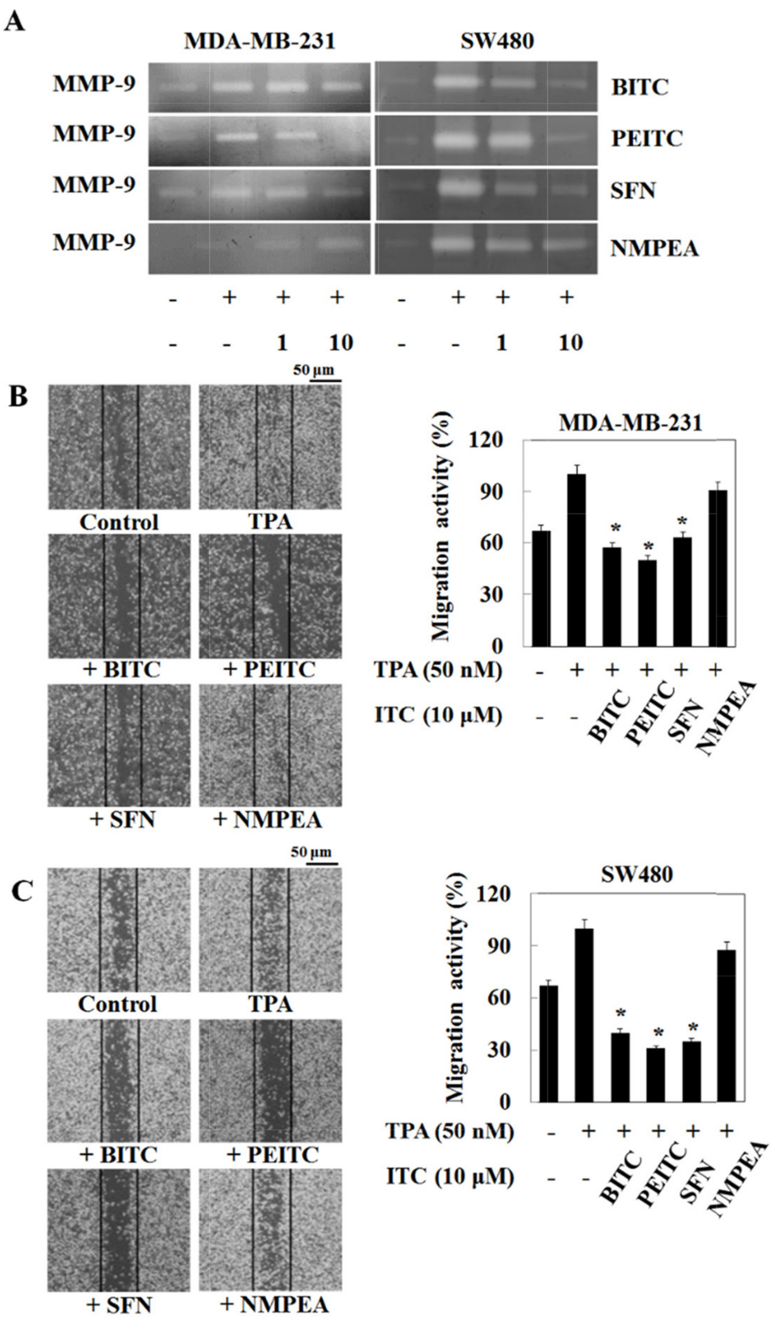

Supplementary Figure 2: (A) Cells were treated with isothiocyanates followed by TPA in serum-free medium. MMP-9 activity was analyzed by a zymography assay. **(B** and **C)** Cells on a dish were scratched and then treated with isothiocyanates followed by TPA for 24 h. Migrating cells were photographed by phase contrast microscopy. The data represent mean ± S.E. of three independent experiments. *$p<0.05$ vs. TPA.

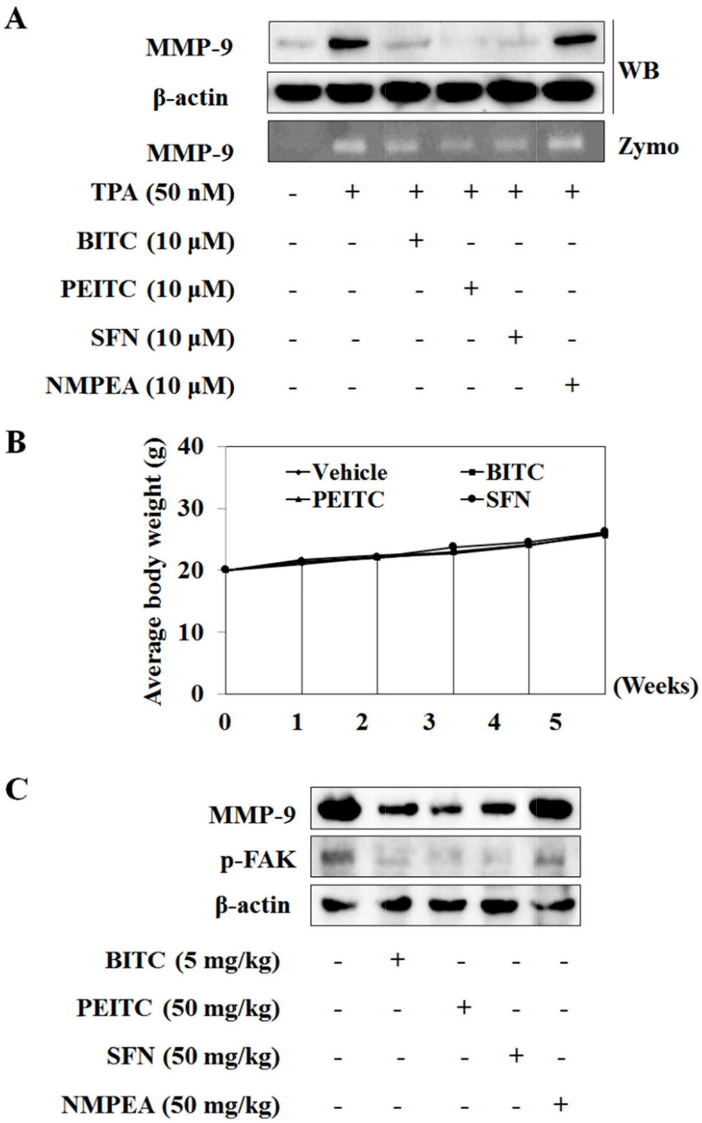

Supplementary Figure 3: (A) Phoenix A-transfected A549 cells were treated with 50 nM TPA in presence of isothiocyanates for 24 h, and the activity and expression of MMP-9 and β-actin as a control were analyzed with zymography and Western blot. **(B)** The mice were inoculated with 1×7^{10} phoenix A-transfected A549 cells subcutaneously into the right flank. After the formation of palpable tumors (~5 mm by day 14), the mice were randomized into five Groups (n=4). The mice were intraperitoneally injected with or without 5 mg/kg/100 µl per mouse BITC, PEITC, and SFN every day. Body weight changes of mice during the 35 days of treatment. **(C)** The expression of MMP-9 and phosphorylation of FAK in vehicle- and isothiocyanate-treated tumor tissue were determined by Western blotting. β-actin is shown as a control. The data represent three independent experiments.
